# The application value and limitations of metagenomic detection technology based on cerebrospinal fluid samples in suspected central nervous system infection: a retrospective study

**DOI:** 10.3389/fmicb.2025.1689253

**Published:** 2026-01-07

**Authors:** Qiujuan Feng, Baoyi Liu, Huazhao Liu, Yaqin Fan, Shanshan Gao, Jia Zhang, Yingjie Kuang, Wenshan Wang, Huiqiang Liang, Yulan Qiu, Huamei Wen, Zize Feng, Yanming Huang, Wanli Zuo, Xin Zhang, Jincheng Zeng, Jinhua Wu, Yuanhao Liang, Jiyong Gu

**Affiliations:** 1 Department of Clinical Laboratory, Jiangmen Central Hospital, Jiangmen, China; 2 Clinical Experimental Center, Jiangmen Engineering Technology Research Center of Clinical Biobank and Translational Research, Jiangmen Key Laboratory of Precision and Clinical Translation Medicine, Jiangmen Central Hospital, Jiangmen, China; 3 Department of Neurology, Jiangmen Central Hospital, Jiangmen, China; 4 Department of Critical Care Medicine, Jiangmen Central Hospital, Jiangmen, China; 5 Department of Hematology, Jiangmen Central Hospital, Jiangmen, China; 6 Department of Neurosurgery, Digital Neurosurgery Operations Center, Jiangmen Central Hospital, Jiangmen, China; 7 Department of Pulmonary and Critical Care Medicine, Jiangmen Central Hospital, Jiangmen, China; 8 Dongguan Key Laboratory of Medical Bioactive Molecular Developmental and Translational Research, Guangdong Provincial Key Laboratory of Medical Immunology and Molecular Diagnostics, Guangdong Medical University, Dongguan, China; 9 Collaborative Innovation Center for Antitumor Active Substance Research and Development, Guangdong Medical University, Zhanjiang, Guangdong, China

**Keywords:** central nervous system infections, cerebrospinal fluid, conventional testing, culture, metagenomics next generation sequencing

## Abstract

**Background:**

Accurately diagnosing central nervous system (CNS) infections remains challenging. This study aimed to evaluate the effectiveness of metagenomic next-generation sequencing (mNGS) in diagnosing suspected CNS infections and its role in facilitating rapid and accurate pathogen identification.

**Methods:**

This retrospective study enrolled cerebrospinal fluid specimens from 246 patients with suspected CNS infections and 20 controls with definitively ruled-out infections. Using clinical diagnoses established by an expert panel based on comprehensive criteria as the reference standard, we evaluated the diagnostic performance of mNGS relative to culture and conventional tests. Additionally, we analyzed the therapeutic guidance value of positive mNGS results and risk factors for false negatives.

**Results:**

mNGS showed 73.2% (180/246) agreement with clinical diagnosis, superior to culture (54.1%, 133/246) and conventional methods (61.4%, 151/246). For general bacteria and fungi, mNGS showed 61.9% (26/42) concordance with culture. False negatives in mNGS predominantly involved viral missed detection. Age, presence of systemic infection, headache, and cerebrospinal fluid glucose levels were likely key determinants of mNGS performance. mNGS detection of Epstein–Barr virus, Streptococcus spp., *Mycobacterium tuberculosis* complex, herpes simplex virus type 1, and Staphylococcus spp. suggested high pathogenic potential, whereas Torque teno virus detection more likely indicated carriage or experimental contamination.

**Conclusion:**

mNGS holds significant value for the diagnosis, therapeutic management, and prognostic assessment of suspected CNS infections.

## 1 Introduction

Central nervous system (CNS) infections, including meningitis, encephalitis, and abscesses, represent life-threatening clinical emergencies associated with high mortality rates, largely due to challenges in pathogen identification (Cain et al., 2019). Conventional diagnostic methods—such as cerebrospinal fluid culture, polymerase chain reaction (PCR) assay, and antigen/antibody detection—are limited by inadequate sensitivity, slow turnaround time, and narrow pathogen coverage, leaving nearly half of cases without a definitive etiology and impeding targeted therapy (Yu et al., 2022).

Metagenomic next-generation sequencing (mNGS) has emerged as a crucial tool in pathogen detection due to its comprehensive coverage and high sensitivity (Wilson et al., 2018; Gu et al., 2019; Ramachandran and Wilson, 2020). Recent investigations have further validated its exceptional diagnostic performance in suspected CNS infections, demonstrating an enhanced capacity to identify pathogens frequently missed by conventional approaches (Cao et al., 2025; Ferreira et al., 2025). We previously established the value of mNGS for pathogen identification and clinical management in infectious diseases (Zhang P. et al., 2020; Zhang et al., 2022).

Nevertheless, key challenges persist regarding the optimal integration of mNGS findings with clinical presentations, particularly in identifying patient-specific factors affecting test performance and developing individualized diagnostic and treatment strategies. While Benoit et al. systematically evaluated mNGS performance in CNS infections through a seven-year retrospective analysis, their examination of false-negative results primarily addressed technical parameters, with limited exploration of clinical risk factors influencing detection efficacy (Benoit et al., 2024).

Therefore, this study evaluates the diagnostic performance of mNGS, with a focus on analyzing the actual impact of positive results on clinical treatment and systematically investigating the clinical risk factors leading to false-negative outcomes, thereby providing a foundation for its precise clinical application.

## 2 Methods

### 2.1 Study design

Retrospective analysis of 285 cases with suspected CNS infection admitted to Jiangmen Central Hospital from June 2022 to March 2024. According to the screening criteria, 39 cases were excluded, and 246 cases were ultimately included.

Inclusion criteria for suspected CNS infection: (1) patients with symptoms of meningitis or encephalitis or imaging suggestive of possible encephalitis meningoencephalitis (detailed criteria for suspected CNS infection are provided in Supplementary Method 1); (2) Administration of empirical therapy for CNS infection prior to mNGS testing. Exclusion criteria: (1) refusal of lumbar puncture or the existence of contraindications to lumbar puncture; (2) patients with missing clinical data; (3) Unable to make a definite diagnosis.

The study received approval from the Ethics Committee of Jiangmen Central Hospital (No: 2022–127), and all participants signed an informed consent form.

### 2.2 Collection of clinical information and diagnostic criteria

Clinical data, imaging examinations, blood inflammatory testing, pathogen detection outcomes and turnaround time (TAT, defined as the entire duration from sample submission to the issuance of the official microbial detection report) from patients were collected. The pathogen detection methods were classified into four categories: (1) mNGS; (2) culture (specific culturable microorganisms are provided in Supplementary Method 2); (3) conventional testing (including CSF smear, culture, serological testing, tissue biopsy, and nucleic acid amplification testing, specific tests are provided in Supplementary Method 3); (4) combined methods (mNGS combined with conventional testing).

The clinical diagnosis was established by three neurologists with associate senior titles or higher, independently applying a pre-defined composite clinical-microbiological reference standard. This standard mandatorily integrated clinical presentation, CSF analysis, imaging findings, conventional microbiological test results (including culture, targeted PCR, broad-range 16S/18S rRNA sequencing, serology, and antibody indices), and response to therapy (van de Beek et al., 2016). Based on this standard, cases were classified into two categories: CNS infection and non-CNS infection. Experts made independent diagnoses after reviewing all information, with a final diagnosis requiring consensus from at least two experts. Comprehensive criteria for the final diagnosis are provided in Supplementary Method 4.

The impact of positive mNGS results on clinical management was assessed by an independent adjudication panel comprising two neurologists and one infectious disease specialist who were not involved in the original patient care. The panel members, blinded to the mNGS reports and initial clinical decisions, reviewed all clinical data and categorized the influence of mNGS into the following four classifications: (1) Guidance on medication use: mNGS provides pathogenetic evidence for unidentified infections, playing an active role in guiding clinical treatment. This includes: (i) Clinicians first identified the pathogen through mNGS before routine test results were reported; (ii) When routine tests were negative or empirical diagnosis was inconclusive, clinicians solely determined the pathogen via mNGS; (iii) Clinicians corrected prior empirical diagnoses and adjusted treatment regimens based on mNGS results. (2) Verify judgment: mNGS validated clinical assessments when its results aligned with physicians’ infection judgments (made via culture/other tests prior to mNGS reporting). (3) Contamination: mNGS results contradicted clinical manifestations, suggesting potential contamination or viral carrier status, thereby interfering with clinical decision-making. (4) Undetermined: without a conclusive determination regarding the pathogenicity of the mNGS result.

### 2.3 Collection and processing of specimens

Lumbar puncture for CSF collection was performed after the initiation of empirical antimicrobial therapy. CSF was obtained under aseptic conditions by a licensed physician. A 1–2 mL aliquot was preserved in nuclease-free tubes, frozen at −20 °C, and processed for mNGS within 48 h of collection. Another 5 mL portion was inoculated into blood culture bottles (bioMérieux, France) and cultured using the BacT/ALERT 3D automated system, with positive cultures identified by matrix-assisted laser desorption/ionization time-of-flight mass spectrometry (MALDI-TOF MS), and the remaining specimen was forwarded for routine CSF examination, smear analysis, and biochemical assessment. Cell biopsies and nucleic acid amplification assays are conducted when necessary. Nucleic acid amplification tests encompass standard PCR and Xpert MTB (for *Mycobacterium tuberculosis* detection).

### 2.4 mNGS detection process

#### 2.4.1 Sample pre-processing and nucleic acid extraction

DNA and RNA were co-extracted in parallel from a single sample following a single freeze–thaw cycle. After thawing at room temperature, 600 μL of CSF was mechanically lysed with glass beads (6.0 M/S, 10 cycles of 1 min 10 s with 10 s intervals) and DNA was extracted using a column-based method. A separate 300 μL aliquot was used for RNA extraction via a magnetic bead-based method in the presence of RNase inhibitors, followed by reverse transcription into cDNA. Nucleic acid extraction and reverse transcription were performed using commercial kits from Guangzhou Weiyuan Medical Devices Co., Ltd. (DNA Extraction Kit: VM002-50; RNA Extraction Kit: VM006-50; Non-Pre Transcriptome Library Construction Kit (ILM) RS and VMRS0013-50R). An internal process control (*Arabidopsis thaliana*) was spiked into each sample prior to extraction to assess nucleic acid recovery efficiency. For quality control, one negative control (NC; sterile nuclease-free water) was included per batch and processed identically to clinical samples throughout the entire workflow (from nucleic acid extraction to sequencing) to establish a comprehensive contaminant background profile (Supplementary Table 2).

#### 2.4.2 Library preparation and sequencing

The extracted DNA and cDNA were used for separate library preparation. Libraries were constructed by enzymatic fragmentation, end-repair, and purification. The final libraries had an insert size of approximately 200–300 bp. After quantification and quality control, the DNA and cDNA libraries from the same sample were pooled in equimolar amounts for sequencing. High-throughput single-end sequencing (75 bp) was performed on the Illumina NextSeq DX500 platform, generating a minimum of 15 million raw reads per sample.

#### 2.4.3 Bioinformatics analysis

Sequencing data were processed using a standardized bioinformatic pipeline with clearly specified versions of all core tools to ensure reproducibility. First, Illumina’s official software bcl2fastq (v2.20.0) was used to convert raw sequencing data into FASTQ files for individual samples. Next, quality control was performed using fastp (v0.23.1), with strict criteria to filter high-quality reads: the Q30 score was required to be at least 85% for qualifying data, each sample needed a minimum of 15 million raw reads, and a read-length filter was applied—only reads ≥50 bp were retained, while low-quality reads, adapter reads, and reads shorter than 50 bp were removed. After quality control, the remaining high-quality reads were aligned to the human reference genome (GRCh38/hg38) using BWA-MEM (v0.7.17) to identify and exclude human sequences. The average percentage of human reads mapped to the human reference genome was 95% (IQR: 89.1–97%).

The non-human reads remaining after human sequence exclusion were then aligned in an end-to-end mode to a custom-built microbial reference genome database (IDseqDB v3.0.0), which was curated in-house to prioritize clinical relevance to central nervous system (CNS) infections. This database was compiled from NCBI RefSeq and GenBank, encompassing 12,142 bacterial, 10,061 viral, 2,680 fungal, 206 mycobacterial, 654 parasitic, and 120 mycoplasma/chlamydial genomes—each selected for its known association with CNS infections. To maintain accuracy, the database is updated quarterly, and its performance is validated against a panel of well-characterized positive clinical specimens. Through this alignment, multiple key evaluation parameters were generated: species-specific matched sequence number (SMRN), standardized SMRN (SDSMRN, SMRN normalized to 20 million total reads), genome coverage (defined as the percentage of the pathogen’s reference genome covered by mapped reads, with a focus on coding regions for bacteria), and sequencing depth. All these parameters were then analyzed by professionals with combined expertise in microbiology and clinical practice.

#### 2.4.4 Interpretation of mNGS data

Suspected pathogens were identified by comparing sample data with the NC; species detected in both were considered contaminants and discarded. Positivity was determined using pre-established, taxon-specific thresholds that integrated read counts, genome coverage, and duplicate library consistency. For viruses, positivity required SMRN ≥3, coverage of ≥3 non-overlapping genomic regions, and detection in ≥1 duplicate library. For bacteria, parasites, and mycoplasma, the threshold was an SDSMRN ratio ≥10 (SDSMRN ratio = SDSMRN of CSF sample / SDSMRN of NC; if NC SDSMRN = 0, the ratio equals the sample’s SDSMRN), combined with genome coverage ≥0.5% and detection in ≥1 duplicate library. For low-biomass pathogens like the *Mycobacterium tuberculosis* complex, rickettsiae, and syphilis spirochetes, a lower threshold of SMRN ≥1 and genome coverage ≥0.1% was used, with detection in ≥1 duplicate library; the same threshold was applied to fungi. A graphical scheme illustrates the stepwise workflow (Supplementary Figure 1).

### 2.5 Definition of pathogen detection consistency

The concordance between mNGS and culture results was evaluated using pre-defined categories. The outcomes of the pathogen consistency assessment were classified as follows: (1) Positive consistency: Pathogen identification was considered positively consistent if the set of pathogens detected by mNGS served as either an identical or an expanded set that included all pathogens identified by culture. (2) Negative consistency: Both mNGS and culture are negative. (3) Overall consistency: Positive consistency and negative consistency. (4) Inconsistency: The results were deemed inconsistent when the pathogens identified by the two methods differed and did not meet the criteria for positive consistency.

### 2.6 Statistical analysis

All data were analyzed using GraphPad Prism 9.3 software (GraphPad Software Inc), R4.3.1 software (The R Foundation). Normally distributed measures were presented as mean ± standard deviation ( $\bar{x}$ ± s), skewed measures as median with interquartile range [M (P25, P75)], and counts as frequency and percentage [*N* (%)]. Comparisons between groups of continuous variables were conducted using the unpaired t-test or the Mann–Whitney U test. Comparisons among groups of categorical variables were conducted with the chi-square test. Logistic regression analysis was employed for correlation assessment. Heat maps were generated utilizing R language software (ggplot package). The diagnostic test used the clinical diagnosis made by three clinicians based on comprehensive judgment criteria as the reference diagnostic criteria, and the Agreement, Positive agreement, Negative agreement, positive predictive value and negative predictive value of different diagnostic methods were calculated. Clinicians’ diagnoses were classified into CNS infection caused by different pathogens and non-CNS infection. When the pathogen detected by a test method was the same as the clinician’s diagnosis of pathogen infection, the result was considered Positive agreement; when a test method was negative and the clinical diagnosis was non-CNS infection, the result was considered Negative agreement. *p* < 0.05 (two-sided) was considered a statistically significant difference.

## 3 Results

### 3.1 General information of patients

**Figure 1:**
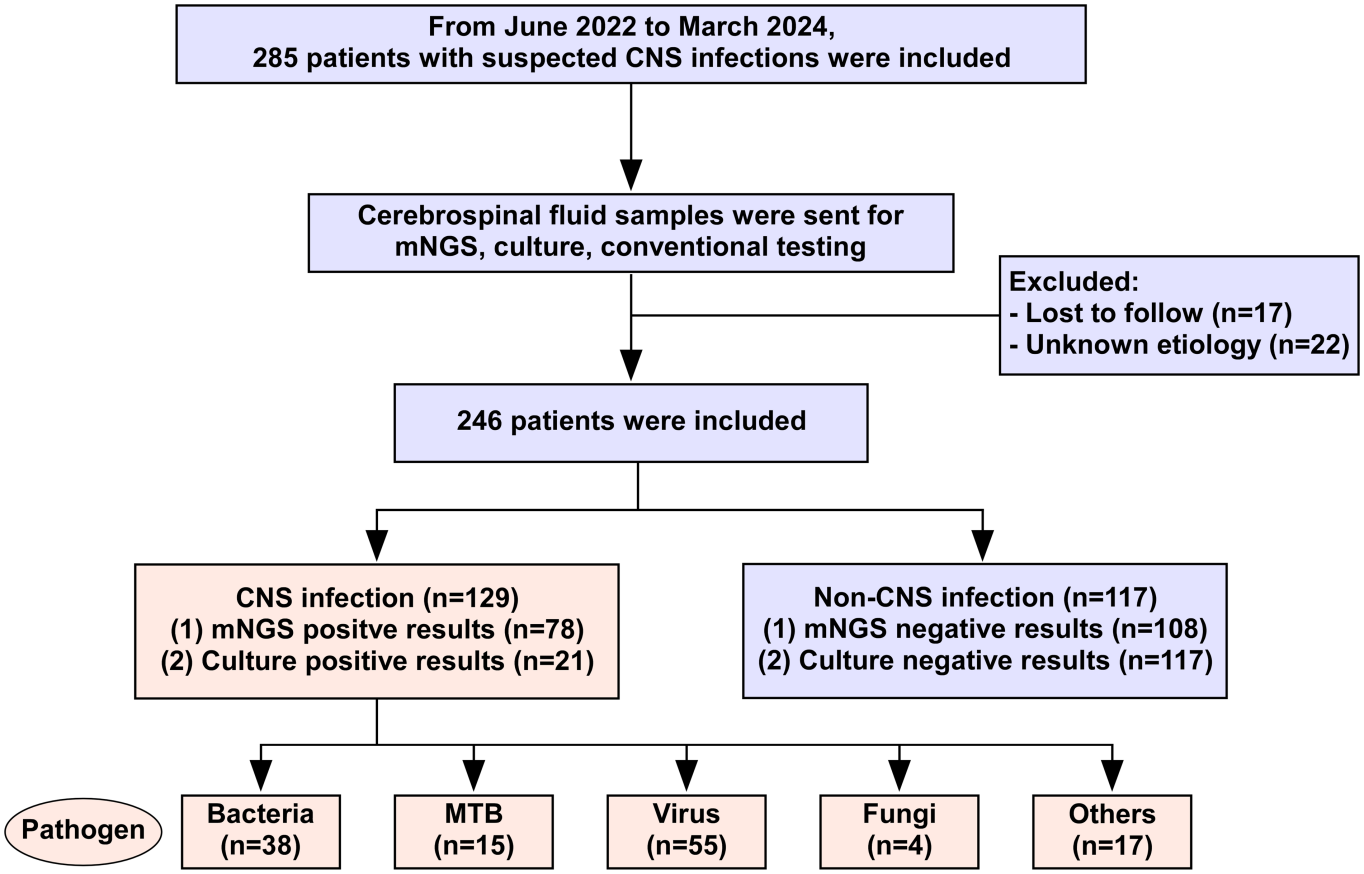
Flowchart for enrolment.

This study analyzed a total of 246 patients with suspected CNS infections. Among them, 129 were diagnosed with CNS infections, 117 with non-CNS infections were designated (Figure 1). Table 1 demonstrates that patients with CNS infections exhibit more significant clinical manifestations and more severe alterations in CSF biochemical parameters compared to those with non-CNS infections (*p* < 0.05).

**Table 1:** Clinical characteristics of patients with suspected CNS infections.

Characteristic	CNS infection (*n* = 129)	Non-CNS infection (*n* = 117)	*p*-value
Age	53 (31, 67)	54 (38, 65)	0.426
Gender			
Male	89 (69)	74 (63.2)	0.342
Female	40 (31)	43 (36.8)	
Clinical symptom			
Positive acute course	108 (83.7)	77 (65.8)	0.001
Fever	100 (77.5)	71 (60.7)	0.004
Headache	50 (38.8)	39 (33.3)	0.376
Vomiting	38 (29.5)	18 (15.4)	0.009
Convulsions	24 (18.6)	27 (23.1)	0.388
Mental behavioral abnormalities	28 (21.7)	15 (12.8)	0.112
Weak in body and limbs	27 (21.3)	37 (31.6)	0.124
Disturbance of consciousness	54 (41.9)	53 (45.3)	0.587
Post-cranial	27 (20.9)	27 (23.1)	0.685
TB history	13 (10.1)	6 (5.1)	0.146
Meningeal irritation sign	43 (34.7)	23 (20.4)	0.014
Pathological reflex	22 (18.2)	13 (11.3)	0.137
Laboratory test			
C-reactive protein	8.74 (1.19, 45.7)	11.1 (0.19, 97.28)	0.376
WBC of whole blood (×10^9^/L)	9.04 (6.2, 11.97)	9.03 (6.86, 11.04)	0.826
Neutrophil percentage	75.2 (66.25, 85.9)	76.7 (65.85, 84.1)	0.986
Positive Pandy tests	67 (52.8)	35 (29.9)	< 0.001
Turbidity of CSF	38 (30.2)	22 (18.8)	0.040
Intracranial pressure (mmH_2_O)	155 (115, 200)	167 (120, 220)	0.354
CSF WBC (×10^6^/L)	47 (8, 340)	3 (1, 22)	< 0.001
CSF RBC	27 (2, 239)	13 (2, 239)	0.111
CSF glucose (mmol/L)	2.82 (1.84, 3.55)	3.55 (3.06, 4.53)	< 0.001
CSF Chlorine (mmol/L)	123.05 (117.4, 127.8)	125.3 (120.65, 127.1)	0.118
CSF Protein (mg/L)	893 (517.5, 1707)	467 (309.3, 973.5)	< 0.001

CNS, central nervous system; TB, tuberculosis; WBC, white blood cell count; RBC, red blood cell count; CSF, cerebrospinal fluid.

### 3.2 Consistency evaluation of mNGS and other methods with clinical diagnosis

**Figure 2:**
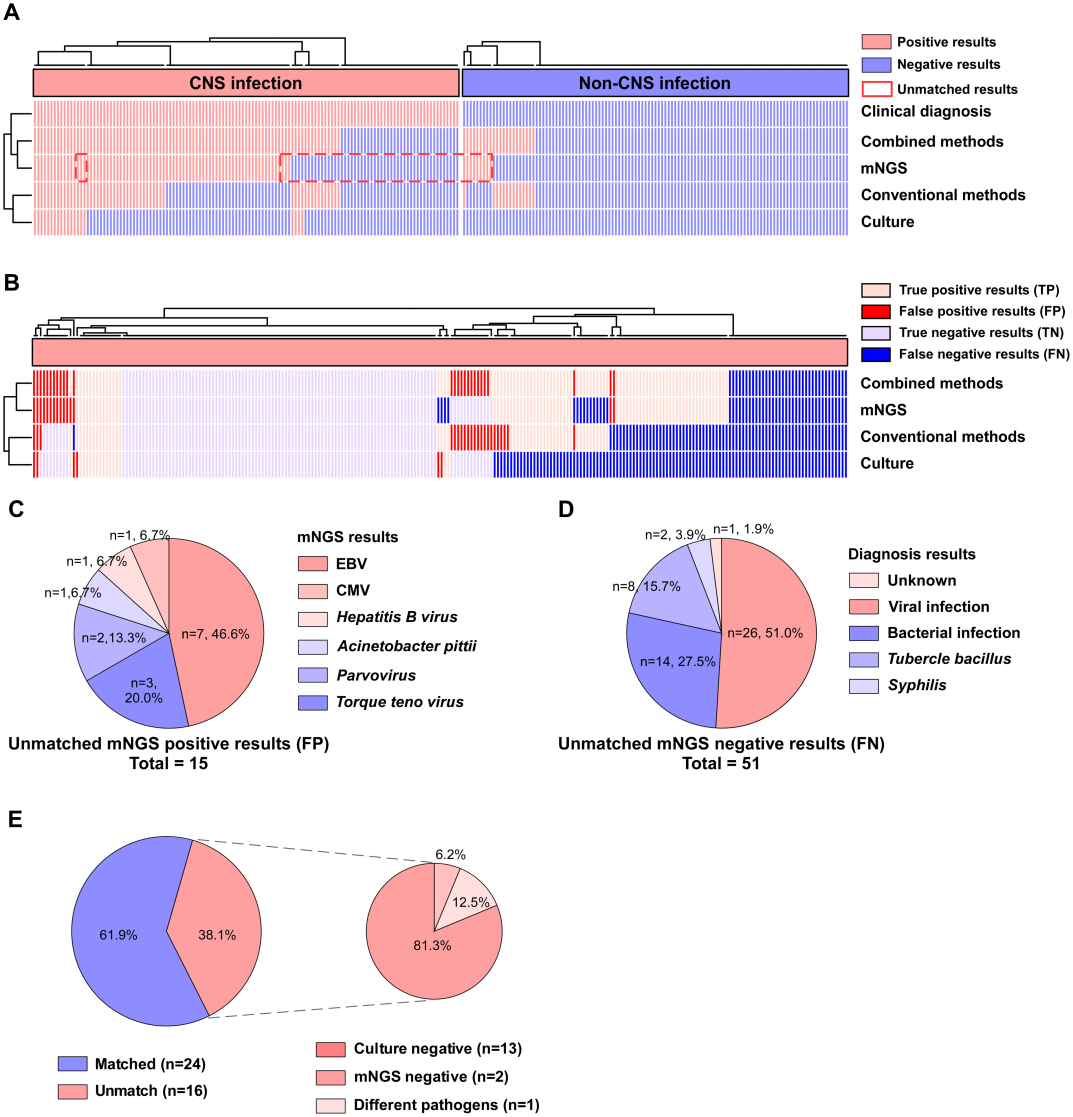
Comparison of diagnostic performance of mNGS, culture, conventional testing, and combined methods in patients with suspected CNS infection. **(A)** Overview of positive results of different testing methods. **(B)** Diagnostic performance of different assays assessed by clinical diagnosis. **(C)** Types of infections in cases with mNGS-positive but clinically discordant results. **(D)** Types of infections in cases with mNGS-negative but clinically discordant results. **(E)** Profile of mNGS versus culture concordance evaluation.

Initially, we found that the positive agreement of mNGS and combined methods for detecting pathogens was notably high, at 58.5% (72/123) and 71.0% (88/124), respectively. Nonetheless, mNGS exhibited discrepancies between the identified pathogens and clinical diagnoses, resulting in 15 false-positive outcomes and 51 false-negative outcomes (Figure 2A). Further findings indicated that the reliability of mNGS detection surpassed that of traditional and culture techniques. In contrast, the conventional and culture methods exhibited higher rates of false negatives, at 29.7% (73/246) and 43.5% (107/246), respectively (Figure 2B). Approximately 50% of the false positive results in mNGS were attributed to *Epstein–Barr virus* (EBV), with *Torque teno virus* (TTV) and *Parvovirus* following closely (Figure 2C; Supplementary Table 1). In the analysis of mNGS false negative results, the majority of missed detections were viruses, with bacteria following closely behind (Figure 2D).

Then, the agreement rates of the four methods were analyzed with reference to clinical diagnosis. It was found that the agreement rates with clinical diagnosis of mNGS and combined detection methods could reach 73.2% (180/246) and 74.4% (183/246), respectively. The culture had the lowest accuracy of 54.1% (133/246). The agreement rate between mNGS results and clinical diagnosis was significantly higher than that of the culture group (X^2^ = 21.19, *p* < 0.001). Overall, both mNGS and the combined method showed optimal and comparable agreement with clinical diagnosis, followed by conventional methods and culture (Table 2).

**Table 2:** The comparison of diagnostic performance of different detection methods.

Detection method	TP	FP	TN	FN	Agreement	Positive agreement	Negative agreement	PPV	NPV	TAT (Day)
mNGS	72	15	108	51	0.732	0.585	0.878	0.828	0.679	1.83 (1.05, 2.10)
Culture	16	6	117	107	0.541	0.130	0.951	0.727	0.522	6.86 (5.05, 7.00)
Conventional methods	48	22	103	73	0.614	0.397	0.824	0.686	0.585	4.08 (1.26, 5.28)
Combined methods	88	27	95	36	0.744	0.710	0.779	0.765	0.725	-

TP, true positive; FP, false positive; TN, true negative; FN, false negative; PPV, positive predictive value; NPV, negative predictive value. TAT, turnaround time, defined as the entire duration from sample submission to the issuance of the official microbial detection report.

Through further analysis of 129 cases of CNS infections, the differences in detection performance among various diagnostic methods across different infection types were evaluated. The results demonstrated that mNGS achieved higher positive detection rates across all infection categories compared to culture-based and conventional methods (Table 3). mNGS exhibited favorable positive agreement with the final clinical diagnosis, with rates ranging between approximately 50 and 70% across different infection types. In contrast, although culture methods retained certain detection capability for bacterial infections, their performance was significantly lower for viral infections, tuberculosis, and rare pathogens, with positive agreement rates of only 0 to 6%. Consequently, mNGS showed a marked advantage over other methods in detecting viruses, *Mycobacterium tuberculosis*, mixed infections, and uncommon infections (Supplementary Figure 2).

**Table 3:** The detection rate of pathogens by various methods in 129 cases of CNS infection.

Pathogen	mNGS	Culture	Conventional methods	Combined methods
Overall (*n* = 129)	60.47% (78/129)	13.18% (17/129)	42.64% (55/129)	72.09% (93/129)
Other Bacteria (*n* = 38)	63.16% (24/38)	31.57% (12/38)	57.89% (22/38)	71.05% (27/38)
Virus (*n* = 55)	52.73% (29/55)	N/A	40.0% (22/55)	70.9% (39/55)
Fungi (*n* = 4)	100% (4/4)	50% (2/4)	75% (3/4)	100% (4/4)
*M.tuberculosis* (*n* = 15)	46.67% (7/15)	N/A	26.67% (4/15)	53.33% (8/15)
Others (*n* = 17)*	82.35% (14/17)	17.65% (3/17)	52.94% (9/17)	88.24% (15/17)

CNS, central nervous system; *M. ctuberculosis, Mycobacterium tuberculosis.*

*Others included 6 cases of mixed infections, 7 cases of specific infections and 4 cases with unclear infection types. NA, not available.

### 3.3 Comparison of mNGS with culture results

Due to the constraints of hospital testing conditions, *Mycobacterium tuberculosis,* viruses and specific pathogens could not be cultivated; thereby, this section exclusively covers bacteria and fungi. The results indicated that, across the various infection types, a certain degree of agreement in positive detection between mNGS and culture was observed only for bacterial infections (Cohen’s kappa = 0.24) (Supplementary Table 3). For the combined bacterial and fungal detections, the coincidence rate of pathogen identification between mNGS and culture was 61.9%. The discrepancy between the two tests was mostly attributed to the positive outcomes of mNGS and the negative outcomes of culture, which constituted 81.3% of the inconsistencies (Figure 2E; Table 4).

**Table 4:** Inconsistency between mNGS and culture detection results.

Patient ID	mNGS result	Culture result
8	*Fusobacterium nucleatum; Parvimonas micra; Klebsiella pneumoniae; Streptococcus intermedius*	/
12	*Streptococcus pneumoniae*	/
16	*Legionella cincinnatiensis*	/
18	*Porphyromonas gingivalis; Tannerella forsythia*	/
19	/	*Staphylococcus epidermidis*
23	*Streptococcus pneumoniae;* EBV	/
44	*Citrobacter freundii; Acinetobacter baumannii*	/
51	*Pseudomonas aeruginosa*	/
185	*Acinetobacter pittii*	/
188	*Staphylococcus epidermidis*	/
198	EBV	*Staphylococcus hominis*
211	*Bacillus cereus*	/
223	/	*Staphylococcus capitis*
235	*Staphylococcus lentus*	/
204	*Aspergillus flavus*	/
233	*Cryptococcus neoformans;* EBV	/

EBV, Epstein–Barr virus.

These findings suggest that culture results can serve as a reference for interpreting mNGS clinical reports, with more credibility when culture and mNGS detection results align. In instances of negative culture detection, mNGS serves as a crucial adjunct for clinically excluding CNS infections.

### 3.4 Influence of positive mNGS outcomes on clinical management

**Figure 3:**
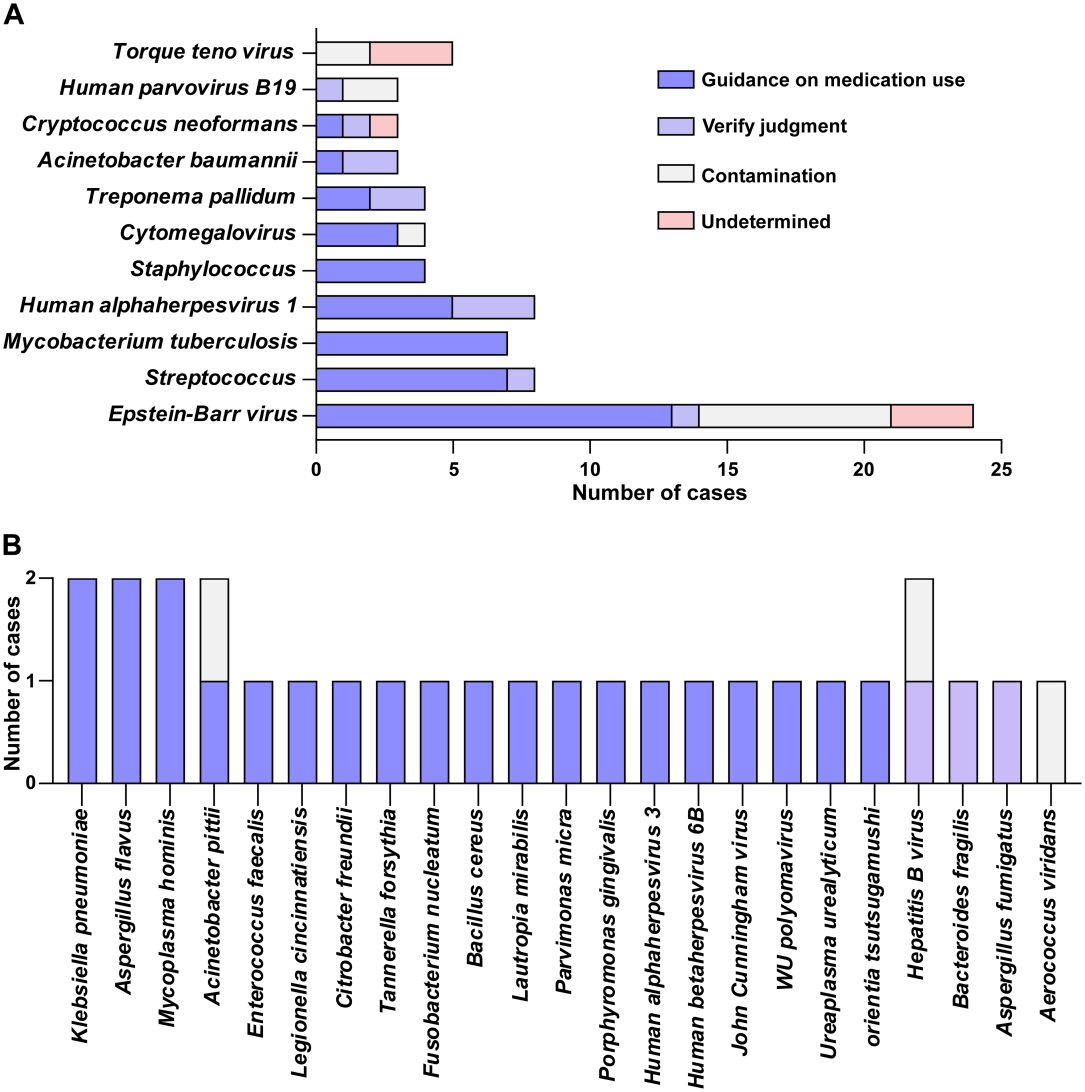
Assessment of the impact of mNGS results on clinical treatment. **(A)** Assessment of the impact of mNGS positive results on clinical care for >3 cases of pathogenic bacteria. **(B)** Assessment of the impact of mNGS positive results on clinical care for ≤ 2 cases of pathogens.

There were 87 cases of positive mNGS results, identifying a total of 41 pathogen species (Figure 3). We categorized the influence of positive mNGS results on clinical treatment into four categories.

The findings indicated an important clinical advantage when the mNGS test yielded positive results, demonstrating its capacity to inform clinical treatment at an early and effective phase. The leading five pathogens linked to clinical medication were EBV, *Streptococcus*, *Mycobacterium tuberculosis complex*, *Human alphaherpesvirus 1*, and *Staphylococcus*. Furthermore, microorganisms whose pathogenicity, viral carrier status, or potential contamination could not be definitively determined were primarily observed with EBV and TTV.

To address the common issue of distinguishing pathogenic infections, sample contamination, or viral carrier status of EBV and TTV in clinical diagnosis, this study conducted supplementary research. Twenty patients definitively excluded from CNS infection (with traumatic brain injury or post-neurosurgical status, and showing no signs of infection) were enrolled as controls, and their CSF samples were collected for mNGS analysis. Additionally, cases clinically diagnosed as CNS infection group and non-infection group were included for comparative analysis, aiming to explore the differences in virus detection rates and sequence number thresholds. The results suggest that the detection of EBV by mNGS is clinically correlated with CNS infections; the detection of TTV may reflect contamination risk during sample processing or a state of viral carriage; relying solely on the sequence abundance of mNGS detection makes it difficult to distinguish pathogenic states (Supplementary Figure 3).

### 3.5 Analysis of risk factors contributing to false-negative mNGS outcomes

**Figure 4:**
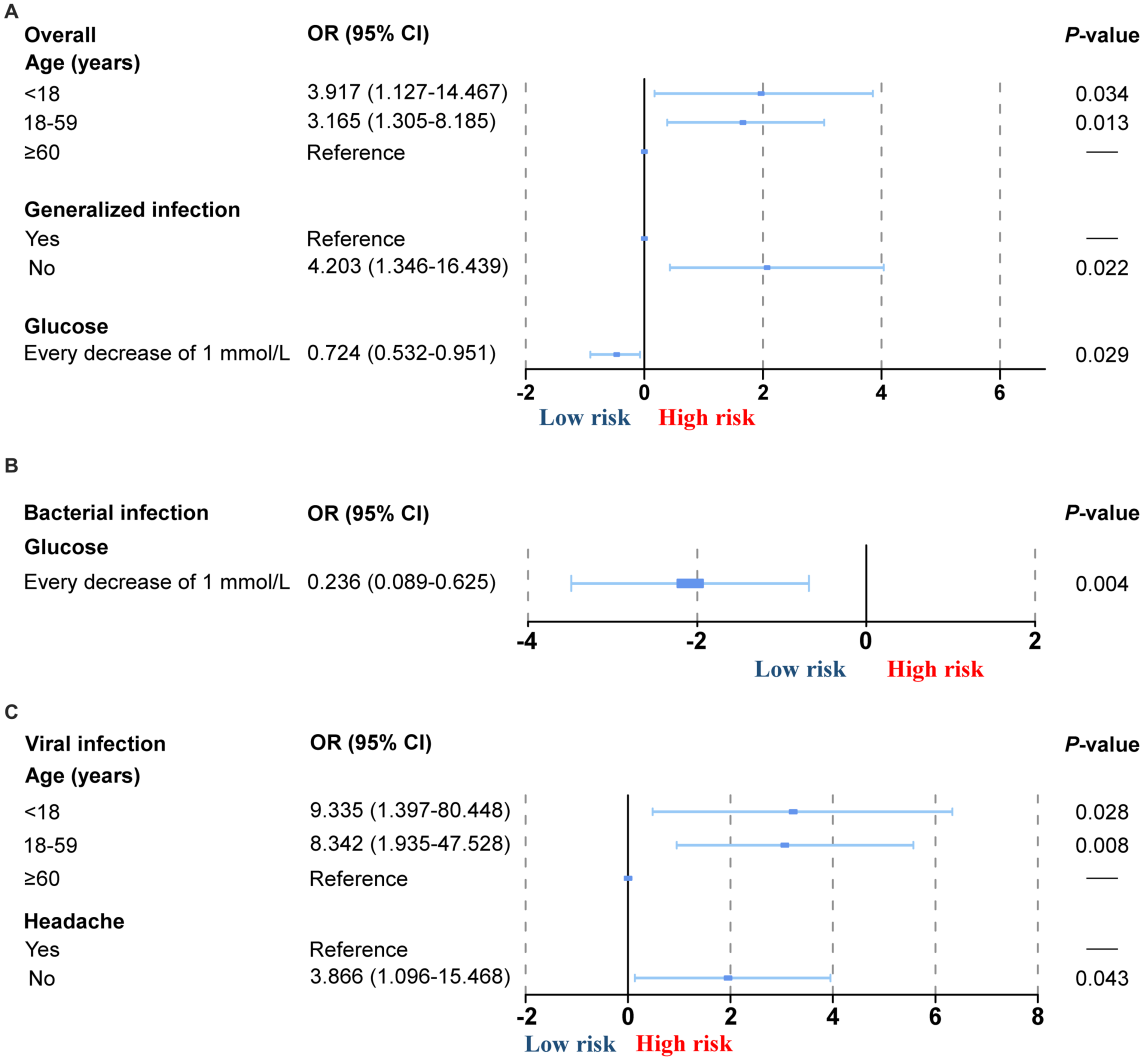
Risk factor analysis for mNGS false-negative results. **(A)** Risk factor analysis for overall mNGS false negative results. **(B)** Risk factor analysis for mNGS false-negative results in patients with viral infections. **(C)** Risk factor analysis for mNGS false-negative results in patients with bacterial infections. The *x*-axis uses a base-2 logarithmic transformation to more clearly represent the relative changes in effect size.

To evaluate potential clinical and laboratory factors associated with false-negative mNGS results, we conducted a comparative analysis between patients with CNS infections who had true-positive mNGS results and those with false-negative mNGS results, as adjudicated by expert clinical diagnosis (Figure 4). The aim was to identify variables that may influence the likelihood of successful pathogen detection by mNGS. Our findings indicated that age, presence of systemic infection, headache symptoms, and CSF glucose levels were significantly associated with false-negative results. Specifically, lower CSF glucose levels were inversely correlated with the likelihood of false-negative outcomes, particularly among patients with bacterial infections (OR = 0.72, *p* < 0.03), suggesting that pronounced inflammatory responses may enhance microbial detectability. Compared to patients aged ≥60 years, those aged 18–59 years and <18 years demonstrated higher odds of false-negative mNGS results. This association was especially evident in the subgroup with viral infections, indicating that younger patients may exhibit lower pathogen loads or atypical presentations, thereby affecting detection sensitivity. The absence of systemic infection was also significantly associated with increased risk of false-negative results (OR = 4.20, *p* < 0.02). In this study, systemic infection was defined as the presence of clinical signs (e.g., fever), elevated peripheral white blood cell count, elevated C-reactive protein, and/or evidence of extracranial infectious foci on imaging or microbiological assessment. Furthermore, among patients with viral infections, the absence of headache was associated with a higher likelihood of false-negative mNGS findings (OR = 3.87, *p* < 0.04). This may reflect limited meningeal involvement or low pathogen concentration in the CSF in patients without typical neurological symptoms. These results suggest that patient age, systemic inflammatory status, neurological symptoms, and CSF biochemical indicators may influence the detection efficiency of mNGS and should be considered when interpreting negative mNGS results in clinically suspected CNS infections.

## 4 Discussion

This study systematically evaluated the value of mNGS in the diagnosis of suspected CNS infectious diseases. The results showed that the positive concordance rate of mNGS with clinical diagnosis was 58.5%, which was in line with the range (45% ~ 70%) reported in the literature (Wilson et al., 2019; Wang J. et al., 2022; Benoit et al., 2024; He et al., 2024).

More importantly, the overall concordance between mNGS and clinical diagnosis was 73.2%, which was significantly better than culture (54.1%) and conventional methods (61.4%), and comparable to the combined methods (both >70%), indicating that mNGS has a high concordance for clinical diagnosis.

Although culture is widely regarded as the “gold standard” for the diagnosis of infectious diseases, its detection rate in this study was low (13.2%, 17/129), with particularly low yields for common community-acquired pathogens such as *Streptococcus pneumoniae* and *Neisseria meningitidis*. This phenomenon is closely related to the real-world clinical context of our study cohort. According to the inclusion criteria, all patients had received empirical antimicrobial therapy prior to mNGS testing, which inevitably significantly reduces the sensitivity of culture (Zhang Y. et al., 2020). Furthermore, low pathogen load, timing of lumbar puncture, and fastidious growth requirements for certain bacteria may have collectively contributed to the negative culture results (Hasbun, 2022). These limitations collectively highlight the critical value of mNGS in establishing an etiological diagnosis following empirical antibiotic treatment.

The property of mNGS to capture microbial nucleic acids without bias provides the advantage of broad-spectrum coverage. In this study, the negative predictive value of mNGS (67.9%) was higher than that of culture (52.2%) and conventional methods (58.5%), suggesting its high reference value for ruling out infection. The above results support the value of mNGS in the diagnosis of suspected CNS infection (Zhang Y. et al., 2020; Liu et al., 2023). Especially when traditional methods are negative, mNGS is a powerful complementary tool for patients with suspected CNS infection.

It was found that the 51 false-negative cases mainly stemmed from missed viral detection, which is consistent with previous studies (Edridge et al., 2019). Viral loads in the cerebrospinal fluid of patients with encephalitis are usually low, viral genomes are small, RNA viruses are easily degraded, and interference with human nucleic acid information in the raw data increases the difficulty of virus detection (Carbo et al., 2020; Jurasz et al., 2021).

We further analysed the clinical factors of missed detection of viral CNS infection pathogens and found that the younger the age of the patient, the probability of missed detection of viral CNS increased. The younger the age of the patient, the clinical presentation is often atypical, especially in neonates (Tyler, 2018), which may affect clinical decision-making and delay the time of delivery, thus affecting the detection rate of mNGS. In terms of clinical symptoms, patients with CNS infection accompanied by headache have a higher rate of mNGS positivity. Headache is usually indicative of meningeal involvement and is more common in patients with meningitis and meningoencephalitis. A study by Benoit et al. (2024) showed a higher rate of mNGS positivity in patients with meningitis and meningoencephalitis than in patients with encephalitis. This may be related to the fact that the infection is confined to the brain parenchyma or that ‘hit-and-run’ exposures result in very transient CNS viraemia. Therefore, in patients with suspected CNS infection who are missing headache symptoms, pathogens tend to be low or absent in the cerebrospinal fluid. For bacterial infections, cerebrospinal fluid glucose levels were negatively correlated with mNGS detection, with lower glucose levels suggesting a more severe infection, in line with the findings of a study (Ait-Ali et al., 2023).

The clinical value of mNGS lies in its capacity for both rapid and precise diagnosis. Our study demonstrated that its median turnaround time (1.83 days) was significantly shorter than that of traditional culture (6.86 days) and conventional methods (4.08 days), providing timely etiological evidence for guiding targeted therapy in 73 patients. In the study, mNGS successfully detected two cases of intracranial infection with *Mycoplasma hominis*, which is a rare central nervous system infection and has only been reported in one case (Dong et al., 2022). In another case of postoperative coma after nasal tumor surgery, the cerebrospinal fluid of a patient was detected by mNGS with a variety of bacteria of oral origin, including *Mycobacterium fragilis* and *Streptococcus pepticus oralis*. In such cases, the detection rate is low and the optimal treatment time is easily delayed due to the long detection period (Zhong et al., 2023), which highlight the key value of mNGS in rapid and accurate diagnosis of difficult infections.

It is worth noting that the mNGS test includes all microbial nucleic acids in the specimen rather than the surviving pathogenic microorganisms, so clinicians need to consider the information of the test report and the patient’s clinical condition when interpreting the report to screen whether it is a pathogenic microorganism. In this study, we found that when mNGS detected *Streptococcus* spp.*, Mycobacterium tuberculosis complex*, HSV-1, and *Staphylococcus* spp., clinicians could give priority to their pathogenicity. As for the detection of viruses such as EBV and TTV, it is difficult for clinicians to differentiate pathogenic infection, sample contamination, or viral carrier status based on mNGS test reports alone.

Currently, there is no unified diagnostic standard for EBV-associated CNS infections. Although the higher detection rate of EBV in the infected group in this study suggests a potential pathogenic association, the interpretation of EBV detection in CSF remains challenging: it could either represent “bystander” reactivation triggered by other pathogenic infections or originate from infected lymphocytes in EBV-associated primary CNS lymphoma (Fan et al., 2019; Wang Y. et al., 2022; Andersen et al., 2023). Therefore, accurate discrimination can rely on a combined testing strategy, namely confirming viral CNS localization and active infection by comparing viral loads (e.g., via qPCR) in paired CSF and plasma samples along with EBV serology (VCA-IgM, EBNA-1 IgG) (Wilson et al., 2019). TTV, as a human commensal virus, its cerebrospinal fluid findings are more consistent with asymptomatic carriage or contamination in the vast majority of cases than with a direct etiology of current CNS infections; although pathogenicity has occasionally been reported in severely immunosuppressed states (Gore et al., 2023), no clear clinical relevance was observed in this study. For CNS latent DNA viruses, higher microbial sequence counts and relative abundance often suggest greater pathogenicity (Schlaberg et al., 2017; Xing et al., 2020). However, this study failed to validate this ‘sequence count threshold’ theory due to the heterogeneity of sample size and viral load. Future studies should combine host immune status, longitudinal viral load monitoring and multi-omics data to establish a hierarchical framework for determining the pathogenicity of latent viruses.

In clinical applications, cerebrospinal fluid mNGS still has several technical challenges, such as sample contamination, low detection rates of intracellular parasites and fungi, difficulty in distinguishing between colonization and infection, and lack of uniform standards for interpretation of results (Chiu and Miller, 2019). However, it still has important application value for early diagnosis and treatment of CNS infectious diseases, improvement of patients’ clinical prognosis and exclusion of non-CNS infectious diseases.

Our study has several limitations. First, its single-center, retrospective design may introduce selection bias. The fact that patients did not undergo identical testing panels may also limit the direct comparability of mNGS with conventional methods. Second, while we sought to minimize incorporation bias by having three senior neurologists establish the clinical diagnosis independently—integrating pathogenetic evidence, imaging findings, and treatment response—the inclusion of mNGS results as part of the reference standard could have influenced the perceived diagnostic performance. Third, the lack of systematically collected paired CSF and plasma samples for viral quantification (e.g., via qPCR) and EBV serology limited our ability to further differentiate between active CNS infection, latent carriage, and contamination of EBV and TTV at the pathogen detection level.

## 5 Conclusion

mNGS has a crucial role in diagnosing, treating, and prognosing suspected CNS infectious diseases, and its integration with traditional methods will enhance the precision of etiologic diagnosis. In summary, mNGS facilitates the swift and precise diagnosis of CNS infections, particularly in cases where pathogens are varied or challenging to identify through traditional techniques. However, its efficacy in detecting viruses remains limited. mNGS results can contribute to clinical diagnosis; yet, it is essential to integrate them with the patient’s clinical characteristics and laboratory data to prevent misleading false-positive or false-negative outcomes.

## Data Availability

The raw data supporting the conclusions of this article will be made available by the authors, without undue reservation.
