# Supplementary material for: The application value and limitations of metagenomic detection technology based on cerebrospinal fluid samples in suspected central nervous system infection: a retrospective study
Source: Front Microbiol. 2026 Jan 7;16:1689253. doi: 10.3389/fmicb.2025.1689253 (PMC12819739; doi:10.3389/fmicb.2025.1689253)
Supplement: Supplementary file 2 [file Supplementary_file_1.docx]

**Supplementary Method 1：Criteria of suspected CNS Infection**

Symptoms and signs of central nervous system infection include one or more of the following: fever (>38℃), headache, meningeal signs, vomiting, convulsions, focal neurological deficits, altered consciousness, or lethargy.

And at least one of the following should be met:

a. Increased white cell count, elevated protein, and/or decreased glucose level in CSF.

b. Cerebral imaging indicates pathologic infection changes.

**Supplementary Method 2：Microorganisms covered by cerebrospinal fluid culture**

| Bacteria |  |  |
| --- | --- | --- |
| GramPositive Bacteria | Cocci | *Staphylococcus spp.* |
|  |  | *Streptococcus spp.* |
|  |  | *Enterococcus spp.* |
|  | Bacilli | *Listeria spp.* |
|  |  | *Bacillus spp.* |
|  |  | *Corynebacterium spp.* |
|  |  | *Lactobacillus spp.* |
|  |  | *Actinomyces spp.* |
|  |  | *Bifidobacterium spp.* |
| GramNegative Bacteria | Cocci | *Neisseria spp.* |
|  |  | *Moraxella spp.* |
|  | Bacilli | *Escherichia spp.* |
|  |  | *Salmonella spp.* |
|  |  | *Shigella spp.* |
|  |  | *Klebsiella spp.* |
|  |  | *Enterobacter spp.* |
|  |  | *Serratia spp.* |
|  |  | *Proteus spp.* |
|  |  | *Citrobacter spp.* |
|  |  | *Edwardsiella spp.* |
|  |  | *Pseudomonas spp.* |
|  |  | *Acinetobacter spp.* |
|  |  | *Stenotrophomonas spp.* |
|  |  | *Alcaligenes spp.* |
|  |  | *Haemophilus spp.* |
|  |  | *Pasteurella spp.* |
| Fungi | Yeasts | *Cryptococcus spp.* |
|  |  | *Candida spp.* |
|  |  | *Rhodotorula spp.* |
|  |  | *Trichosporon spp.* |
|  | Molds | *Aspergillus spp.* |
|  |  | *Fusarium spp.* |
|  | Dimorphic fungi | *Histoplasma spp.* |
|  |  | *Coccidioides spp.* |

**Supplementary Method 3：Routine Testing Items**

1. CSF Smear
2. CSF Acid-Fast Stain
3. CSF India Ink Stain
4. CSF Culture
5. Cell Biopsy
6. EBV Antigen/Antibody Test
7. HIV Antigen/Antibody Test
8. Treponema pallidum Antigen/Antibody Test
9. Hepatitis C Virus (HCV) Antibody Test
10. Hepatitis B Virus (HBV) Antigen/Antibody Test
11. Influenza A Virus Antigen Test
12. Influenza B Virus Antigen Test
13. Cytomegalovirus (CMV) Antibody Test
14. Herpes Simplex Virus (HSV) Antibody Test
15. Widal-Weil Test
16. Fungal (1,3)-β-D-Glucan Test
17. Influenza A Virus Nucleic Acid Test
18. Influenza B Virus Nucleic Acid Test
19. EBV Nucleic Acid Test
20. Cytomegalovirus (CMV) Nucleic Acid Test
21. Herpes Simplex Virus (HSV) Nucleic Acid Test
22. Mycobacterium tuberculosis Complex Nucleic Acid Test
23. Aspergillus fumigatus Nucleic Acid Test
24. Aspergillus flavus Nucleic Acid Test
25. Aspergillus niger Nucleic Acid Test
26. Candida albicans Nucleic Acid Test
27. Candida tropicalis Nucleic Acid Test
28. Candida glabrata Nucleic Acid Test
29. Staphylococcus aureus Nucleic Acid Test
30. Methicillin-Resistant Staphylococcus (MRS) Nucleic Acid Test
31. Klebsiella pneumoniae Nucleic Acid Test
32. Pseudomonas aeruginosa Nucleic Acid Test
33. Streptococcus pneumoniae Nucleic Acid Test
34. Acinetobacter baumannii Nucleic Acid Test

**Supplementary Method 4：Composite criteria of final diagnosis of CNS infection**

Central nervous system infection: Clinical criteria with or without etiology criteria.

Non-central nervous system infection: Clinical criteria was not met.

1. Clinical criteria: Symptoms and signs of central nervous system infection include one or more of the following: fever (>38℃), headache, meningeal signs, vomiting, convulsions, focal neurological deficits, altered consciousness, or lethargy.

And at least one of the following should be meet:

a. Increased white cell count, elevated protein, and/or decreased glucose level in CSF

b. Cerebral imaging indicates pathological infection changes

c. Recovered by effective treatment

2. Etiology criteria: At least one of the following methods should be met:

a. There is a positive CSF culture of pathogenic microbes.

b. Targeted PCR detection

c. Serology and antibody indices

d. Broad-range 16S/18S rRNA sequencing
