# Supplementary material for: The application value and limitations of metagenomic detection technology based on cerebrospinal fluid samples in suspected central nervous system infection: a retrospective study
Source: Front Microbiol. 2026 Jan 7;16:1689253. doi: 10.3389/fmicb.2025.1689253 (PMC12819739; doi:10.3389/fmicb.2025.1689253)
Supplement: Supplementary file 3 [file Supplementary_file_2.docx]

**Supplementary table 1** Inconsistency between positive mNGS results and clinical diagnoses

| Patient ID | Pathogens considered in clinical diagnosis | Pathogens detected by mNGS |
| --- | --- | --- |
| 24 | Unknow etiology | *Human parvovirus B19* |
| 31 | Unknow etiology | *Human parvovirus B19* |
| 163 | Bacteria | *Cytomegalovirus* |
| 26 | Bacteria | EBV |
| 198 | *Staphylococcus hominis* | EBV |
| 200 | *Mycobacterium tuberculosis* | EBV |
| 109 | Non-CNS infection | *Acinetobacter pittii* |
| 126 | Non-CNS infection | *Hepatitis B virus* |
| 80 | Non-CNS infection | *Human parvovirus B19* |
| 81 | Non-CNS infection | *Human parvovirus B19* |
| 108 | Non-CNS infection | *Human parvovirus B19* |
| 83 | Non-CNS infection | EBV |
| 65 | Non-CNS infection | EBV |
| 141 | Non-CNS infection | EBV |
| 254 | Non-CNS infection | *Aerococcus viridans,* EBV |

EBV, *Epstein-Barr virus.*

**Supplemental table 2** Contaminant profile of negative controls (NCs).

| **Category** | **Microorganism** |
| --- | --- |
| **Environmental Bacteria** |  |
| Gram-negative | *Acinetobacter johnsonii* |
|  | *Moraxella osloensis* |
|  | *Acinetobacter lwoffii* |
|  | *Chryseobacterium hominis* |
|  | *Pseudomonas aeruginosa* |
| Gram-positive | *Micrococcus luteus* |
|  | *Microbacterium aurum* |
|  | *Rhodococcus erythropolis* |
|  | *Gordonia sputi* |
| **Skin Flora** |  |
| Gram-positive | *Cutibacterium acnes* |
|  | *Corynebacterium falsenii* |
|  | *Staphylococcus epidermidis* |
|  | *Staphylococcus hominis* |
|  | *Cutibacterium granulosum* |
|  | *Staphylococcus warneri* |
|  | *Propionibacterium namnetense* |
| **Oral Flora** |  |
| Gram-positive | *Corynebacterium matruchotii* |
| **Fungus** | *Malassezia restricta* |

This table lists contaminants that were present in at least two-thirds of the negative control samples. These microorganisms are regarded as common background in the experimental workflow and should be critically evaluated when detected in clinical specimens.

**Supplemental table 3** Comparison of mNGS vs. Culture for positive detection.

|  | mNGS+  /Culture+ | mNGS+  /Culture- | mNGS-  /Culture+ | mNGS-  /Culture- | McNemar‘s P-value | Cohen’s κ (95% CI) |
| --- | --- | --- | --- | --- | --- | --- |
| Overall (n=129) | 13 (10%) | 59 (46%) | 7 (5%) | 50 (39%) | 0.368 | 0.053 (0-0.166) |
| Bacteria (n=38) | 9 (24%) | 12 (32%) | 3 (8%) | 14 (37%) | 0.161 | 0.24 (0-0.510) |
| Virus (n=55) | 1 (2%) | 28 (51%) | 2 (4%) | 24 (44%) | 0.598 | -0.04 (NA) |
| Fungi (n=4) | 2 (50%) | 2 (50%) | 0 (0%) | 0 (0%) | >0.999 | 0 (0-0) |
| M.tuberculosis (n=15) | 0 (0%) | 6 (40%) | 0 (0%) | 9 (60%) | >0.999 | 0 (0-0) |
| Others (n=17)* | 1 (6%) | 11 (65%) | 2 (12%) | 3 (18%) | 0.191 | -0.208 (NA) |

*Others included 6 cases of mixed infections, 7 cases of specific infections and 4 cases with unclear infection types. NA, not available.
