# Supplementary material for: The application value and limitations of metagenomic detection technology based on cerebrospinal fluid samples in suspected central nervous system infection: a retrospective study
Source: Front Microbiol. 2026 Jan 7;16:1689253. doi: 10.3389/fmicb.2025.1689253 (PMC12819739; doi:10.3389/fmicb.2025.1689253)
Supplement: Supplementary file 4 [file Supplementary_file_3.docx]

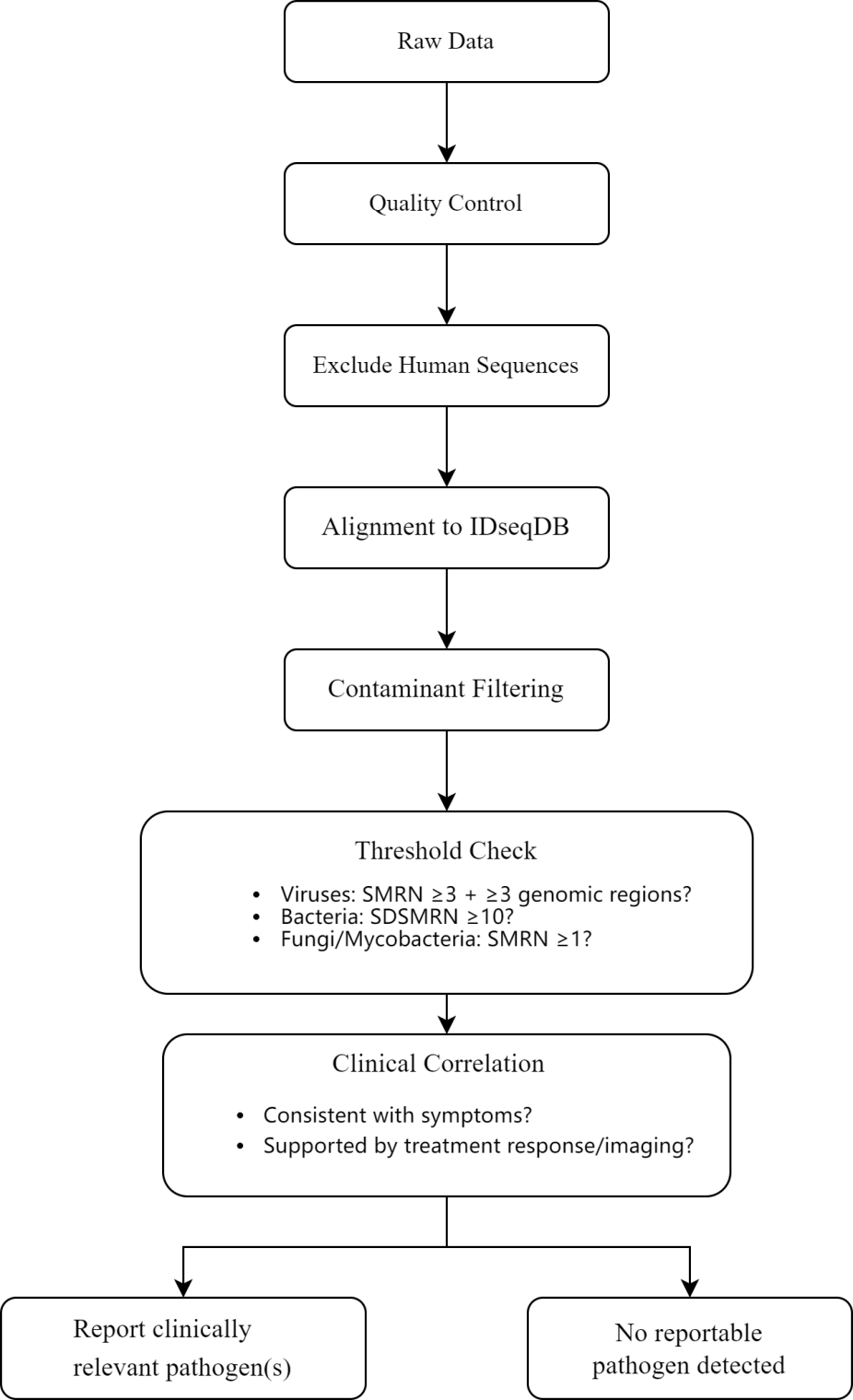


**Supplemental figure 1 Decision workflow for mNGS pathogen identification.** Starting with quality control and human sequence exclusion, followed by microbial alignment and calculation of key parameters (SMRN, SDSMRN, genome coverage, and sequencing depth—the average number of reads covering each base in the pathogen’s reference genome), then contaminant exclusion via control comparisons, next verification against a priori thresholds and duplicate library consistency, and finally validation with clinical context (patient symptoms, imaging results, and treatment response). Additionally, for each batch of samples, the full contaminant profile of the extraction blank was reported, including a list of detected contaminants, their SMRN/SDSMRN values in the blank, and the exclusion criteria used (pathogens with sample SDSMRN ≤ blank SDSMRN were excluded). Ultimately, an mNGS result was considered positive only if it was consistent with the detected microorganism’s pathogenicity, the patient’s clinical characteristics, and their response to treatment—ensuring that results were both microbiologically valid and clinically relevant.

*
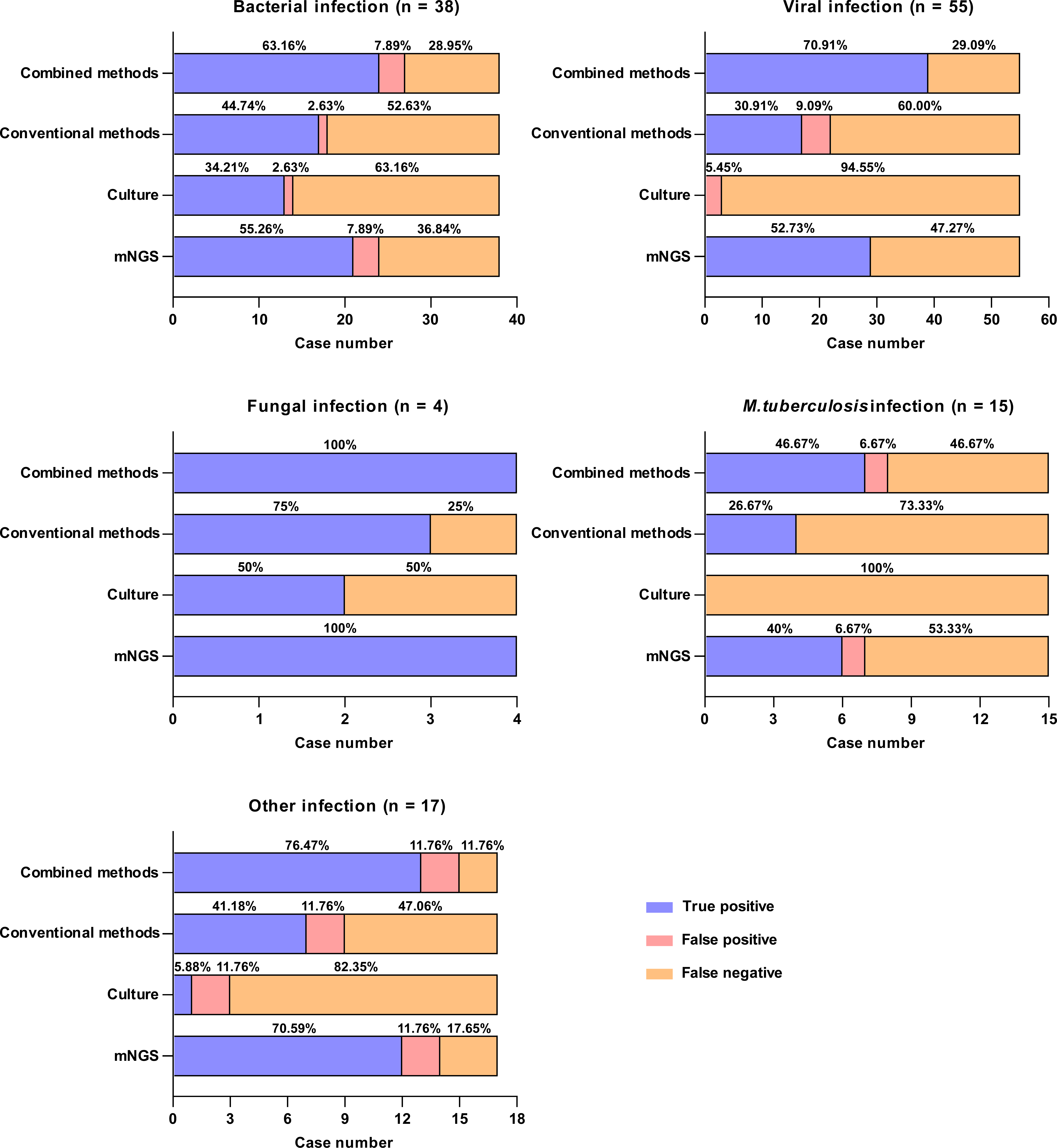
*

**Supplemental figure 2 Evaluation of diagnostic performance across different methods in 129 confirmed CNS infection cases across distinct infection subtype subgroups.**


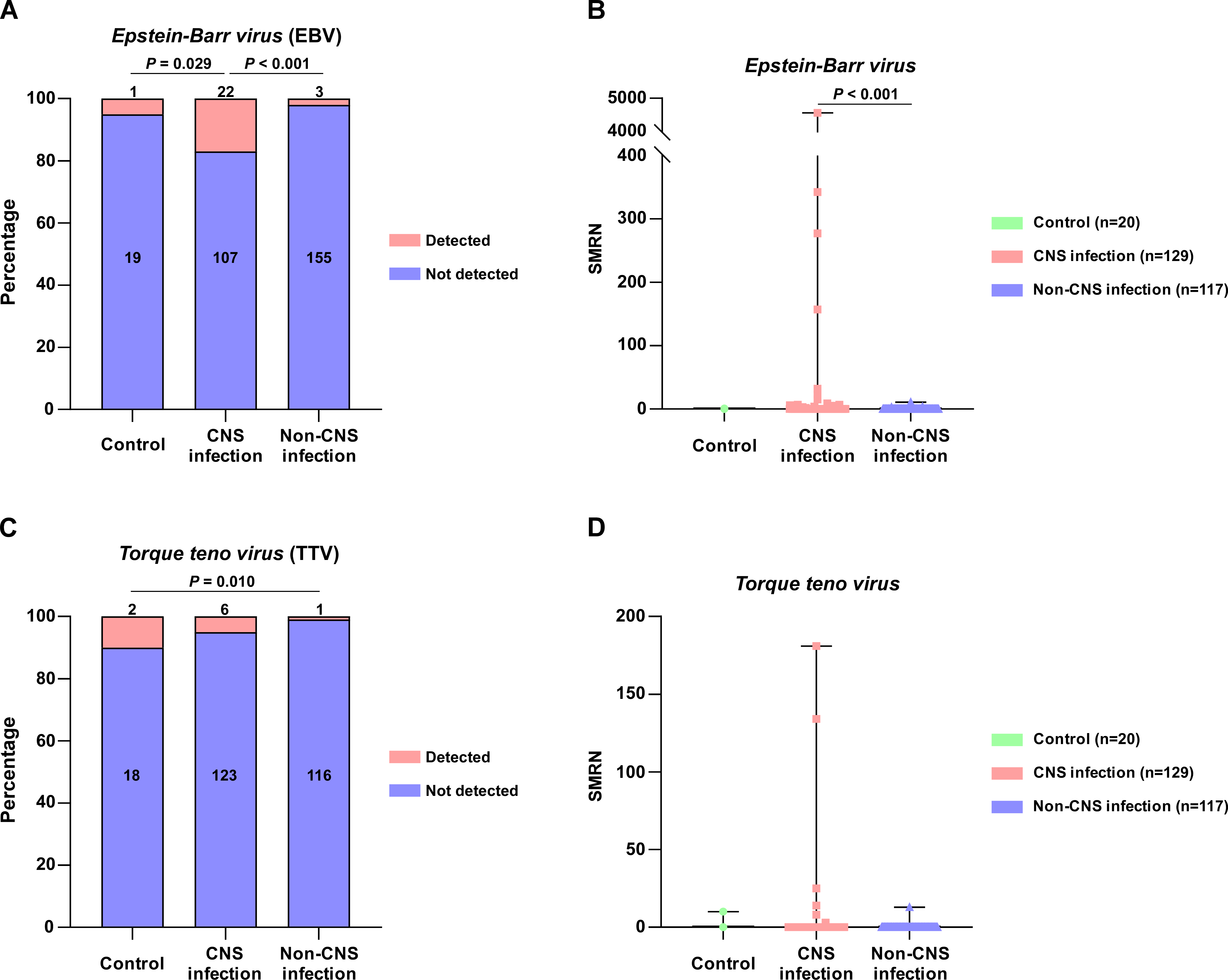


**Supplemental figure 3 Comparison of detection of EBV and TTV in three groups.** (A) Comparison of EBV detection, SMRN ≥1 is considered to be detectable. (B) Comparison of SMRN of EBV. (C) Comparison of TTV detection. (D) Comparison of SMRN of TTV. SMRN, species-specific matched sequence number.
